# Preoperative Nomogram for Differentiation of Histological Subtypes in Ovarian Cancer Based on Computer Tomography Radiomics

**DOI:** 10.3389/fonc.2021.642892

**Published:** 2021-03-25

**Authors:** Haiyan Zhu, Yao Ai, Jindi Zhang, Ji Zhang, Juebin Jin, Congying Xie, Huafang Su, Xiance Jin

**Affiliations:** ^1^ Department of Gynecology, Shanghai First Maternal and Infant Hospital, Tongji University School of Medicine, Shanghai, China; ^2^ Department of Gynecology, The 1st Affiliated Hospital of Wenzhou Medical University, Wenzhou, China; ^3^ Department of Radiotherapy Center, The 1st Affiliated Hospital of Wenzhou Medical University, Wenzhou, China; ^4^ Department of Medical Engineering, The 1st Affiliated Hospital of Wenzhou Medical University, Wenzhou, China; ^5^ Department of Radiation and Medical Oncology, The 1st Affiliated Hospital of Wenzhou Medical University, Wenzhou, China; ^6^ Department of Radiation and Medical Oncology, The 2nd Affiliated Hospital of Wenzhou Medical University, Wenzhou, China

**Keywords:** ovarian cancer, epithelial ovarian cancer, non-epithelial ovarian cancer, computed tomography, radiomics, nomogram

## Abstract

**Objectives:**

Non-invasive method to predict the histological subtypes preoperatively is essential for the overall management of ovarian cancer (OC). The feasibility of radiomics in the differentiating of epithelial ovarian cancer (EOC) and non-epithelial ovarian cancer (NEOC) based on computed tomography (CT) images was investigated.

**Methods:**

Radiomics features were extracted from preoperative CT for 101 patients with pathologically proven OC. Radiomics signature was built using the least absolute shrinkage and selection operator (LASSO) logistic regression. A nomogram was developed with the combination of radiomics features and clinical factors to differentiate EOC and NEOC.

**Results:**

Eight radiomics features were selected to build a radiomics signature with an area under curve (AUC) of 0.781 (95% confidence interval (CI), 0.666 -0.897) in the discrimination between EOC and NEOC. The AUC of the combined model integrating clinical factors and radiomics features was 0.869 (95% CI, 0.783 -0.955). The nomogram demonstrated that the combined model provides a better net benefit to predict histological subtypes compared with radiomics signature and clinical factors alone when the threshold probability is within a range from 0.43 to 0.97.

**Conclusions:**

Nomogram developed with CT radiomics signature and clinical factors is feasible to predict the histological subtypes preoperative for patients with OC.

## Highlights

1. The differentiation of histological subtypes is critical for the assessment of the prognosis and treatment responses of patients with ovarian cancer (OC);
2. Radiomics features derived from preoperative CT images alone or combing with clinical factors were investigated to predict the histological subtypes to help physician to optimize the management for patients with OC and achieved an area under curve (AUC) of 0.869;
3. The present study showed the feasibility of the CT radiomics signature combining with clinical factors for predicting the histological subtypes of OC. A nomogram was constructed to be used clinically to assess histological types for individual OC patients preoperatively.

## Introduction

Ovarian cancer (OC) is the deadliest form of gynecological malignancy, which consists of approximately one fourth of all the gynecological cancers but with a cancer-associated mortality approximately the combined rates of cervical and uterine cancers (1). The emerging of targeted therapy and identification of gene abnormalities in different histological subtypes open new perspectives for a personalized management for patients with OC (2, 3). The differentiation of histological subtypes is critical for the assessment of the prognosis and treatment responses of cancer patients (4, 5). Pathologically, OC is divided into two subtypes: Epithelial ovarian cancer (EOC) and non-epithelial ovarian cancer (NEOC) (6). EOC accounts for approximately 85-90%, while NEOC accounts for about 10% of OC (7).

There is a significant difference in the therapeutic schedule for EOC and NEOC treatment. For example, some subtypes of EOC such as clear cell and mucinous ovarian cancers which resistant to conventional platinum/taxane chemotherapy due to the differences in chemosensitivity (8–10). Another, fertility sparing treatment should be under consideration in patients with NEOC as it is frequently found in young childbearing women, in spite of the NEOC represents a small group of gynecological cancers (11). Consequently, an accurate identification of histological types in patients with OC in preoperative is important since it guides the personalized treatment and surveillance planning.

Currently, surgery or tissue biopsy (cytopatholgy) is usually applied to differentiate OC (12). Frozen section diagnosis following surgery is an important and helpful method for the diagnosis and classification of EOC and NEOC (13). However, the invasive nature of surgery diagnosis and biopsy bring additional risks and cost for patients. In addition, biopsy with fine-needle aspiration is not recommended for some early-stage OC to avoid rupturing the cyst and spilling malignant cells into the peritoneal cavity (14, 15). On the other hand, the results of surgical specimen and biopsy may be affected by the heterogeneity of tumor, especially for large ovarian masses (16). Thus, an accurate, non-invasive method to predict the histological subtypes preoperatively is essential for the overall management of OC (17).

In clinical setting, medical imaging demonstrates strong prognostic value with the ability to visualize a cancer’s appearance on a macroscopic level noninvasively, and is routinely applied to detect and characterize OC (18, 19). Due to the superior advantages of wide availability, high cost-efficiency, fast image scanning, and good reproducibility, computed tomography (CT) is recommended by the European Society of Urogenital Radiology and the American College of Radiology as the standard imaging method for preoperative and postoperative assessment of women with OC (20, 21). The main limitation of CT images is the low sensitivity and specificity resulted from its low soft tissue contrast (22). Furthermore, the reliability of CT assessment is also limited by the variety of experience of operators and radiologists (23, 24).

Radiomics is an emerging technique that transforms digital medical images into mineable high-dimensional data by extracting quantitative features mathematically (25). Radiomics features may help to characterize tumor biology *in vivo* by correlating these features with ground truth pathology diagnosis (26). Recently, radiomics signature has proven to be a significant classification biomarker for lung cancer and brain metastasis histological subtypes (27, 28). CT radiomics has also been investigated for the identification of benign and malignant tumors and for the prediction of clinical outcomes for patients with OC (29, 30). However, there is still no quantitative approach for distinguishing of EOC and NEOC noninvasively. The purpose of this study is to investigate the feasibility and accuracy of radiomics signature in the differentiating of EOC and NEOC based on CT images for patients with OC.

## Materials and Methods

### Patients’ Selection

**Figure 1:**
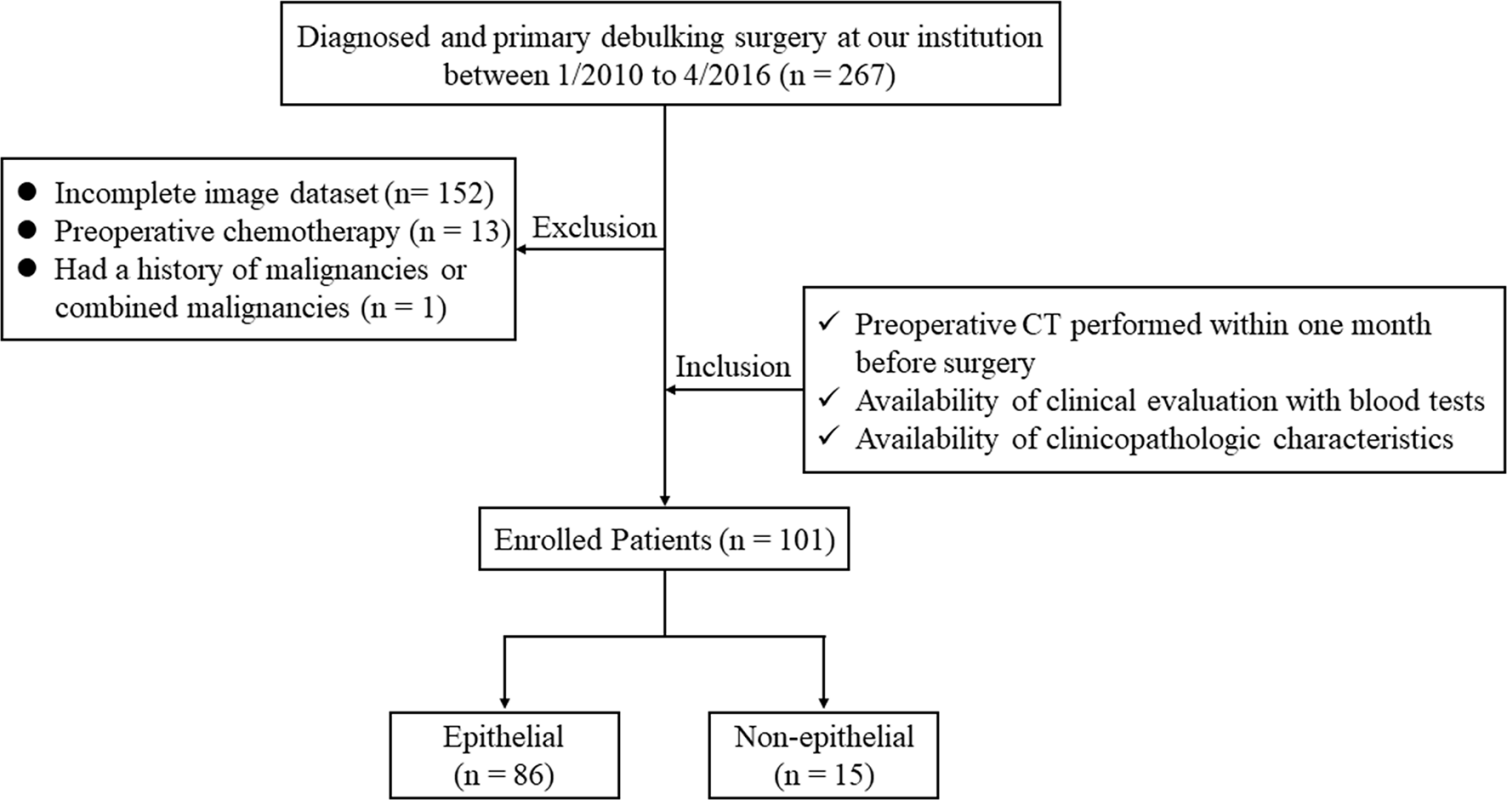
The flowchart of the case identification process.

Ethical approval for this retrospective study was obtained from the Institutional Review Board of our hospital and conducted in accordance with the Declaration of Helsinki (ECCR no. 2019059). Informed consent was waived by ECCR for the retrospective nature of this study. By searching the electronic medical records, a total of 267 patients who underwent primary debulking surgery with a diagnosis of OC at our hospital between January 2010 and April 2016 were retrospectively reviewed. The inclusion criteria were as follows: (I) patients underwent routine, unenhanced CT examination within one month before surgery; (II) available with routine clinical evaluation of blood tests; and (III) available with clinicopathologic characteristics, including age, weight, International Federation of Gynecology and Obstetrics classification (FIGO) stage, and histological subtypes. The exclusion criteria were as follows: (I) lack of digital imaging data (*n* = 152); (II) treated with preoperative chemotherapy (*n* = 13); and (III) with a history of other malignancies or combined malignancies (*n* = 1). Consequently, 101 patients with OC were enrolled in our study and were divide into an epithelial group (*n* = 86) and non-epithelial group (*n* = 15). The flowchart of the case identification process was shown in **Figure 1**.

### CT Images Acquisition and Tumor Segmentations

All the preoperative non-contrast enhanced CT images were acquired with one of the following CT scanners: Bright Speed (GE Healthcare, Milwaukee, WI, USA), or Brilliance (Philips Healthcare, Cleveland OH, USA). The scanning parameters were given below: 120kV, auto tube current, rotation time of 0.4 or 0.5 s, a field of view of 300-500 mm, a pixel size of 512 × 512, a slice interval and thickness of 5 mm with a reconstructed section thickness of 3 mm. All CT images were retrieved from the picture archiving and communication system (PACS).

Tumor volumes were manually segmented by a radiologist with 7 years of experience in gynecological imaging using LIFEx package (http://www.lifexsoft.org) (31). All the segmentations were confirmed by a senior radiologist with over 15 years of experience in gynecological imaging. Further radiomics feature extraction was carried out on the delineated tumor volumes.

### Radiomics Features Extraction and Model Building

Preprocessing with intensity normalization and spatial resampling were performed for all CT images in LIFEx, which was then used to extract radiomics features. LIFEx has been applied in the image biomarker standardization initiative (IBSI). CT images were resampled to a new spacing of 1mm × 1 mm × 3 mm and an intensity range of 0-255 HU. After normalized, a total of 148 radiomics features were extracted based on different matrices by capturing the spatial intensity distributions at four different scales. There were 23 first-order features derived from histogram, shape and conventional statistics, and 125second-order features derived from gray-level co-occurrence matrix (GLCM), neighborhood gray-level different matrix (NGLDM), gray-level run length matrix (GLRLM), and gray-level zone length matrix (GLZLM), respectively. The details of the radiomics feature calculation were shown in the **Supplementary Doc. S1**.

The selection of key radiomics features was performed with Mann-Whitney *U* tests and the least absolute shrinkage selection operator (LASSO) (32). Features with a p < 0.05 in Mann-Whitney *U* tests were selected as potentially informative features, then, optimal features for histological type prediction were identified using the “elastic net”, which is a combination of LASSO and ridge regression. The parameters of the elastic net were tuned with ten-fold cross validation to reduce the reductant information and to avoid over-fitting. A minimum standard deviation and maximum area under curves (AUC) were achieved by tuning coefficient λ. The final radiomic signature was a linear combination of selected features multiplying by their respective weights, and calculated for each patient.

### Clinical Factors and Model Building

The clinical factors of age, weight, total cholesterol (TCHO) (≤ 5.2 or > 5.2 mmol/L), triglyceride (TG) (≤ 1.7 or > 1.7 mmol/L), high density lipoprotein (HDLC) (≤ 2 or > 2 mmol/L), low density lipoprotein (LDLC) (≤ 3.12 or > 3.12 mmol/L), blood sugar (≤ 6.1 or > 6.1 mmol/L), cancer antigen 125 (CA-125) (≤ 35 or > 35 U/ml), and carcinoembryonic antigen (CEA) (≤ 5 or > 5 ng/ml) were collected. The threshold values for TCHO, TG, HDLC, LDLC, blood sugar, CA-125, and CEA levels were decided based on the normal ranges used at our institute.

Univariate analysis was applied to select the related clinical factors in the prediction of histological subtypes. The difference of clinical variables between epithelial and non-epithelial groups was compared by using the chi-square test or by using the Mann-Whitney *U* test. To evaluate the value of clinical factors in the prediction of histological subtypes, clinical factors with a *p*<0.05 in univariate analysis were selected. A logistic regression model was constructed to predict the histological subtypes by fitting the selected clinical factors. The combined model was constructed by combining the CT-based radiomics signature and clinical factors by using multivariable logistic regression analysis.

### Model Evaluation and Clinical Application

The value of the radiomics signature, clinical model and combined model in predicting histological subtypes were evaluate by receiver operating characteristics (ROC) curves and were compared using DeLong test. The AUCs were calculated along with a 95% confidence interval (CI) to evaluate the accuracy of these models. The goodness-of-fit of combined model was assessed by Nagelkerke R^2^, Akaike Information Criterion (AIC) and Brier score. The lower the AIC value and Brier score means the better of model fits, and the higher Nagelkerke R^2^ indicates better calibration. A nomogram was constructed from the combined model to provide the clinicians and patients an individualized and easy-to use tool for the prediction of the histological subtypes. The nomogram is a visual representation of the combined model which equal levels of prediction performance. The predictors of histological subtypes in the nomogram include the radiomics signature and selected clinical factors. The agreement between the histological subtype predictions and the actual outcomes was assessed using a calibration curve. Besides, the Accuracy (ACC), Specificity (SPE), Sensitivity (SEN), Positive predictive value (PPV) and Negative predictive value (NPV) were used to evaluate the value of combined model for the prediction of the histological subtypes.

Considering the training and validation were performed on the same patient group, which may potentially overestimate the performance of the prediction models, the internal validation by bootstrap resampling techniques was done to optimize the model performance. Each bootstrap sample was derived and applied to the original sample without change. The discriminatory index derived from the bootstrap sample subtract the index from the original sample is an estimate of optimism. An average optimism was obtained across 1000 bootstrap replications, which is subtracted from the discriminatory index of the final model’s fit to obtain the overfitting-corrected estimate. In addition, a Hosmer-Lemeshow test was used to assess the performance of the nomogram (33). To determine the clinical value of the radiomics nomogram, decision curve analysis (DCA) was conducted by quantifying the net benefits at different threshold probabilities in the whole group (34).

### Statistical Analysis

Statistical analysis was performed using R analysis platform (version 3.6.0) andOriginPro2016. The used R packages of this paper are listed in the **Supplementary Table S1**. Categorical variables were compared by using the chi-square test. Continuous variables were compared by using the Mann-Whitney *U* test. For all tests, *p*< 0.05 was considered as statically significant.

## Results

### Patients’ Characteristics

The clinical characteristics of enrolled patients in this study were presented in **Table 1**. The median weight and age of the enrolled 101 patients were 56 kgs (range from 42-81) and 54.23 years (range from 15-79), respectively. Metastasis was found in 71 (70.3%) patients. More than half of the patients (56.4%) were found with stage III. The EOC was found in 86 (85.1%) patients, and 12 (11.9%) presented with vascular invasion.

**Table 1:** Clinical characteristic of patients enrolled with ovarian cancer.

Characteristics	Patients (*n* = 101)
Weight (kgs), mean (range)	56 (42–81)
Age (years), mean (range)	54.23 (15–79)
Patients with metastasis	71 (70.3%)
FIGO stage	
I	28 (27.7%)
II	14 (13.9%)
III	57 (56.4%)
IV	2 (2.0%)
Histological type	
Epithelial	86 (85.1%)
Non-epithelial	15 (14.9%)
Vascular invasion	
Yes	12 (11.9%)
No	89 (88.1%)

FIGO, International Federation of Gynecology and Obstetrics.

### Radiomics Features and Clinical Factors

**Figure 2:**
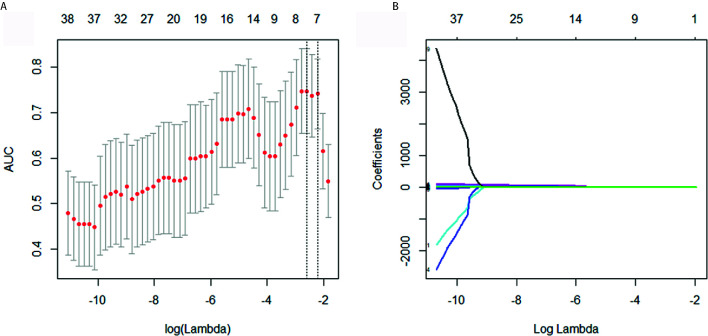
Selection of histological subtypes-associated radiomics features using the elastic net method. **(A)** Tuning parameter (λ) in the elastic net used 10-fold cross validation *via* maximum area under curve and criterion of minimum standard deviation were followed. **(B)** The coefficient profiles of 39 radiomics features. A coefficient profile plot was generated by violating the log (λ) sequence.

Of the 148 radiomics features, 39 were selected according to the Mann-Whitney *U* test with a *p*< 0.05. According to **Figures 2A, B**, eight features were further screened out from the 39 features to build the radiomics signature using the LASSO logic regression model. These features included 1 conventional statistics feature, 3 shape features, and 4grey-level run length matrix (GLRLM) features. The details of the radiomics score calculation formula was shown in the **Supplementary Doc. S2**, and the radiomics score for each patient was calculated. The results of univariate analysis on preoperative clinical factors associated with histological subtypes were presented in **Table 2**. The results indicated that age and CA-125 levels were histological subtype-related factors for patients with OC.

**Table 2:** Univariate analysis of preoperative clinical factors associated with histological subtypes.

Characteristics	Non-epithelial (*n* = 15)	Epithelial (*n* = 86)	*p*
Age (years), Mean (range)	48 (15–74)	58.5 (23–79)	0.008*
Weight (kg) Mean (range)	56 (47–75)	56 (42–81)	0.353
TCHO (mmol/L)			0.569
≤5.2	8	54	
>5.2	7	32	
TG (mmol/L)			0.204
≤1.7	9	66	
>1.7	6	20	
HDLC (mmol/L)			0.386
≤2	14	84	
>2	1	2	
LDLC (mmol/L)			0.254
≤3.12	7	55	
>3.12	8	31	
Blood sugar (mmol/L)			0.594
≤6.1	9	52	
>6.1	6	34	
CA125 (U/ml)			0.002*
≤35	7	9	
>35	8	77	
CEA (ng/ml)			0.219
≤5	9	65	
>5	6	21	

*p value < 0.05; Categorical variables were compared by using the chi-square test. Continues variables were compared by using the Student t test or Mann-Whitney U test;

TCHO, total cholesterol; TG, triglyceride; HDLC, high density lipoprotein; LDLC, low density lipoprotein; CA125, carcinoma antigen 125; CEA, carcinoembryonic antigen.

### Models Performance

**Figure 3:**
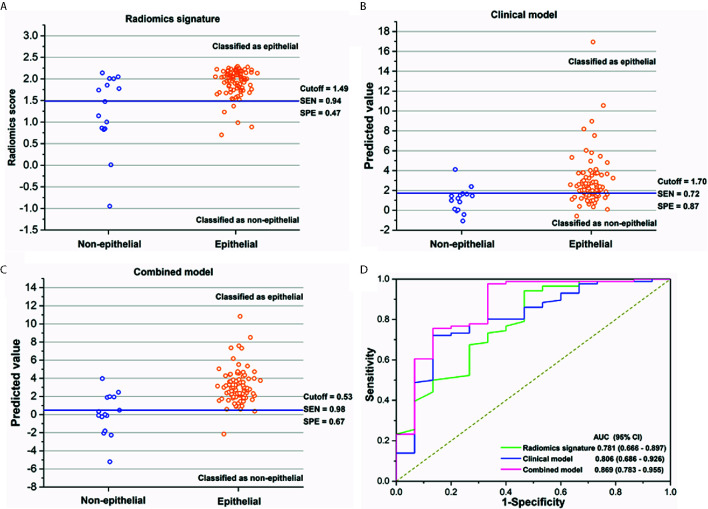
**(A–C)** The performance of the radiomics signature, clinical model, and combined model for the discrimination of histological subtypes in each patient. The blue solid line indicates the best cutoff of the radiomics score or the predicted value of two models for the discrimination of histological subtypes; patients above the cutoff were classified as epithelial group, while patients below the cutoff were classified as non-epithelial group. **(D)** The receiver operating characteristics (ROC) curves with area under curves (AUCs) for radiomics signature, clinical factor model, and combined model of 0.781 (95% CI, 0.666–0.897), 0.806 (95% CI, 0.686–0.926), and 0.869 (95% CI, 0.783–0.955), respectively.

As shown in **Figures 3A–C**, the sensitivity and specificity in the differentiation of EOC and NEOC for radiomics signature, clinical model, and combined model were 0.94, 0.47; 0.72, 0.87; and 0.98, 0.67, respectively. The AUCs of the radiomics signature, clinical model, and combined model were 0.781 (95% CI, 0.666 -0.897) with a cutoff of 1.49, 0.806 (95% CI, 0.686–0.926) with a cutoff of 1.70, and 0.869 (95% CI, 0.783 -0.955) with a cutoff of 0.53, respectively, as shown in **Figure 3D**. The combined model was better than either radiomics signature (0.869 vs 0.781, *p* = 0.02) or clinical model (0.869 vs 0.806, *p* = 0.014) alone. Besides, the combined model exhibited a higher goodness of fit (Nagelkerke R2: 0.45; AIC: 63.12; Brier score: 0.08) and corrected performance (Corrected AUC: 0.84), as shown in **Table 3**.

**Table 3:** Performance of combined model.

	Goodness of fit			Discrimination							Corrected performance
Model	Nagelkerke R^2^	AIC	Brier Score	ACC	SPE	SEN	PPV	NPV	AUC	Internal Validated AUC	
Combined model	0.45	63.12	0.08	0.92	0.67	0.98	0.92	0.82	0.87	0.84	

AIC, Akaike information criterion; ACC, accuracy; SPE, Specificity; SEN, sensitivity; PPV, positive predictive value; NPV, negative predictive value; AUC, area under receiver operating characteristic curve.

Internal validation was performed with 1000 replicate bootstrapping on the primary cohort.

### Nomogram

**Figure 4:**
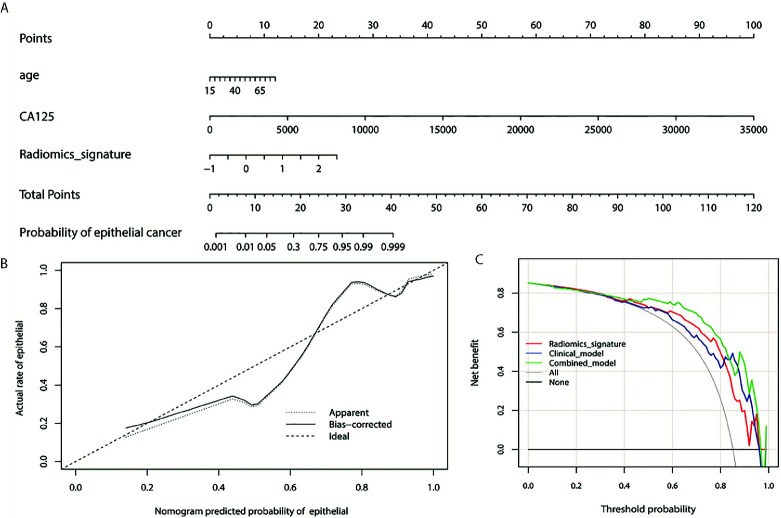
**(A)** Nomogram for the prediction of histological types. The different values for each variable corresponds to a point at the top of the graph, while the sum of the points for all the variables corresponds to a total point, draw a line from the total points to the bottom line is the probability of epithelial. **(B)** the calibration curve of the combined model showing the difference between the predicted probability of histological type and the actual probability. The “Ideal” line represents the perfect prediction as the predicted probabilities equal to the observed probabilities. The “Apparent” curve is the calibration of the entire cohort. The “Bias-correct” curve was the calibration created by internal validation of 1000-replicate bootstrap on the entire cohort. **(C)** the decision curve analysis for the radiomics signature, clinical model, and combined model. On the horizontal axis is the net benefit. The threshold probability is on the vertical axis. The gray line represents the assumption that all patients with epithelial OC. The black line represents the assumption that all patients with non-epithelial OC. The red line represents the radiomics signature, blue line and green line represent the clinical model and combined model respectively.

We enrolled the age, CA125 and radiomics signature as factors in a multivariable logistic regression analysis to build the personalized histological subtypes prediction model. The coefficients and odds ratios of the model are listed in **Table 4**. All factor were discovered as independent risks for histological subtypes prediction. A nomogram was developed based on radiomics features and clinical factors, as shown in **Figure 4**. The calibration curve for the nomogram was tested using Hosmer-Lemeshow test, and showed a nonsignificant statistic (*p*= 0.155). This demonstrates that there is no significant deviation between the calibration curve and a perfect fit for predicting histological type, as shown in **Figure 4B**. The DCA for the radiomics signature, clinical model, and combined model are presented in **Figure 4C**. The combined model provides a better net benefit to predict histological types compared with the other two models when the threshold probability is within a range from 0.43 to 0.97.

**Table 4:** Results of the Multivariable Logistic Regression.

	Coefficient	Odds ratios (95%CI)	*P*
Intercept	−6.008		0.003
Age	0.065	1.068(1.058-1.827)	0.011
CA125	0.010	1.031(1.013-1.128)	0.027
Radiomics signature	2.360	10.593(2.649-60.784)	0.003

## Discussion and Conclusions

The feasibility of CT based radiomics for the differentiation of EOC and NEOC for patients with OC was investigated in this study. Radiomics features combined with clinical factors demonstrated an excellent differentiation accuracy with an AUC of 0.869. Nomogram indicated that the combined model provides a better net benefit in the differentiation of EOC and NEOC compared with radiomics signature and clinical model when the threshold probability is within a range from 0.43 to 0.97.

The mortality of OC is highest among all gynecologic malignancies as approximately two-thirds of cases are diagnosed with advanced stage disease (35). The tumor characteristics and treatment quality were reported as the most important prognostic parameters in the management of OC (5, 36). In this study, EOC consists of 85.1% of the enrolled 101 OC patients. This is consistent with previous reported data that EOC comprises the majority of malignant ovarian neoplasms (85-90%) (37). Serous, endometrioid, mucinous, and clear cell are the four main subtypes of EOC, in which serous histology is the major subtype (about 70%) (38). However, studies indicated that the histologic subtypes of EOC have limited prognostic significance except for clear cell carcinomas (39). Therefore, the differentiation between EOC and NEOC preoperatively is of great clinical value in the management of patients with OC. Subtype classification of Type I and Type II for EOC based on morphologic and molecular heterogeneity was not investigated in this study (40).

Radiomics features had been aggressively investigated as surrogate markers of underlying molecular properties of tumors and used as a noninvasive mean to characterize biologic activities of cancers (41). Quantitative CT features had been investigated for patients with OC to evaluate the associations between tumor heterogeneity and clinical outcomes (30), the association between features and Classification of Ovarian Cancer (CLOVAR) genomic subtypes (42), and to predict the early response of chemotherapy (43). Recently, Zhang et al. classified Type I and Type II EOC based on magnetic resonant imaging (MRI) radiomics features and achieved an accuracy of 0.84 (29). However, few studies have addressed the differentiation between EOC and NEOC with radiomics. In this study, radiomics signature based on preoperative CT images was developed to differentiate EOC and NEOC noninvasively for patients with OC. The AUC achieved by radiomics feature alone was 0.781 with a sensitivity and specificity of 0.94 and 0.47, respectively.

CA-125 has been applied in the screening of OC due to its greater concentrations in OC tumor cells than in other cells of the human body, although the sensitivity and specificity of CA-125 was questioned (44). The feasibility of CA-125 in predicting the likelihood of specific ovarian tumor pathology was reported by Van Calster et al. with limited clinical value (45). In this study, we found that age and CA-125 were correlated with pathological types of OC according to univariate analysis. Model based on age and CA-125 achieved an AUC of 0.806 in the discrimination of EOC and NEOC. An AUC of 0.869 was achieved after combining the radiomics features and clinical factors in this study. This is very close to the overall accuracy of 89.8% achieved by frozen section analysis during intraoperative histopathologic determination (46). DCA of nomogram analysis further verified the good discrimination of combined radiomics features and clinical factors in the differentiation of EOC and NEOC.

One limitation of our study is that this is a retrospective study with a relatively small sample size, where division of training and validation cohorts might cause bias, so the performance of combined model was corrected by internal validation of bootstrap. Therefore, this study can be regarded as an exploratory effort for future external validation on a larger scale. Secondly, only CT image features were investigated in this study. Combining other image modalities, such as ultrasound images or MRI may improve the performance of prediction model. Thirdly, CT images did not include contrast enhanced sequence, which may expand the feature pool and found more valuable radiomics features. In the future, independent validation in larger samples is necessary to improve the confidence and performance of the current model. Finally, the feature reproducibility analysis, such as inter- and intra- observer agreement, as well as the external validation were not performed in this study due to the retrospective nature of the images data and the study.

In conclusion, the present study showed the feasibility of the CT radiomics signature combine with clinical factors for predicting the histological subtypes of OC. A nomogram was constructed to be used clinically to assess histological types for individual OC patients preoperatively.

## Data Availability Statement

The original contributions presented in the study are included in the article/**Supplementary Material**; further inquiries can be directed to the corresponding authors.

## Ethics Statement

This study conformed to the guidelines of the Declaration of Helsinki, and the study has been approved by the Institutional Review Board of The First Affiliated Hospital of Wenzhou Medical University (ECCR no. 2019059).

## Author Contributions

Conception and design: CX, HS, XJ. Administrative support: HZ, YA. Provision of study materials or patients: HZ, YA. Collection and assembly of data: YA, JZ. Data analysis and interpretation: JZ, JDZ, JJ. Manuscript writing: YA, HZ, XJ. Final approval of manuscript: HZ, YA, CX, HF, XJ. All authors contributed to the article and approved the submitted version.

## Funding

This work was partially funded by Wenzhou Municipal Science and Technology Bureau (nos. 2018ZY016, Y20190183, and H20180003) and National Natural Science Foundation of China (no. 11675122).

## Conflict of Interest

The authors declare that the research was conducted in the absence of any commercial or financial relationships that could be construed as a potential conflict of interest.

## Abbreviations

OC, ovarian cancer; CT, computed tomography; LASSO, least absolute shrinkage selection operator; FIGO, International Federation of Gynecology and Obstetrics; EOC, epithelial ovarian cancer; NEOC, non-epithelial ovarian cancer; GLCM, gray-level co-occurrence matrix; NGLDM, neighborhood gray-level different matrix; GLRLM, gray-level run length matrix; GLZLM, gray-level zone length matrix; TCHO, total cholesterol; TG, triglyceride; HDLC, high density lipoprotein; CA125, cancer antigen 125; CEA, carcinoembryonic antigen; ROC, receiver operating characteristics; AUCs, area under the curves; DCA, decision curve analysis.
