# Supplementary material for: Preoperative Nomogram for Differentiation of Histological Subtypes in Ovarian Cancer Based on Computer Tomography Radiomics
Source: Front Oncol. 2021 Mar 25;11:642892. doi: 10.3389/fonc.2021.642892 (PMC8027335; doi:10.3389/fonc.2021.642892)
Supplement: Supplementary file 1 [file DataSheet_1.docx]

**Supplementary Materials**

**Doc. S1: Radiomic feature extraction**

1. **First Order Features**
   1. **Indices from shape**

- 1. **Indices from histogram**

- 1. **Conventional Indices**

$$CONV_{mean}=\frac{1}{N}\sum_{i} Value_{i}$$

$$CONV\_TLG(ml)=V\cdot\frac{1}{N}\sum_{i} SUV_{i}$$

1. **Texture Features**
   1. **GLCM definition**

The grey level co-occurrence matrix (GLCM) takes into account the arrangements of pairs of voxels to calculate textural indices.

- 1. **NGLDM definition**

The neighborhood grey-level different matrix (NGLDM) corresponds to the difference of grey-levels between one voxel and its 26 neighbours in 3 dimensions.

- 1. **GLRLM definition**

The grey-level run length matrix (GLRLM) gives the size of homogeneous runs for each grey-level.

- 1. **GLZLM definition**

The grey-level zone length matrix (GLRLM) provides information on the size of homogeneous zones for each grey-level in 3 dimensions.

$$GLZLM\_SZE=\frac{1}{H}\sum_{i} \sum_{j} \frac{GLZLM(i,j)}{j^{2}}$$

$$GLZLM\_LZE=\frac{1}{H}\sum_{i} \sum_{j} GLZLM(i,j)\cdot j^{2}$$

$$GLZLM\_LGZE=\frac{1}{H}\sum_{i} \sum_{j} \frac{GLZLM(i,j)}{i^{2}}$$

$$GLZLM\_HGZE=\frac{1}{H}\sum_{i} \sum_{j} GLZLM(i,j)\cdot i^{2}$$

$$GLZLM\_SZLGE=\frac{1}{H}\sum_{i} \sum_{j} \frac{GLZLM(i,j)}{i^{2}\cdot j^{2}}$$

$$GLZLM\_SZHGE=\frac{1}{H}\sum_{i} \sum_{j} \frac{GLZLM(i,j)\cdot i^{2}}{j^{2}}$$

$$GLZLM\_LZLGE=\frac{1}{H}\sum_{i} \sum_{j} \frac{GLZLM(i,j)\cdot j^{2}}{i^{2}}$$

$$GLZLM\_LZHGE=\frac{1}{H}\sum_{i} \sum_{j} GLZLM(i,j)\cdot i^{2}\cdot j^{2}$$

$$GLZLM\_GLNUz=\frac{1}{H}\sum_{i} (\sum_{j} GLZLM(i,j))^{2}$$

$$GLZLM\_ZLNU=\frac{1}{H}\sum_{j} (\sum_{i} GLZLM(i,j))^{2}$$

$$GLZLM\_ZP=\frac{H}{\sum_{i} \sum_{j} (j\cdot GLZLM(i,j))}$$

**Doc. S2: Radiomic signature calculation formula**

$$\begin{matrix} Radiomcis score=2.5144+(-0.0025743)*HISTO\_S\text{ke}wness \\ + (-0.0000660679)\cdot*SHAPE\_Volume \\ +(-0.0000002304478)*SHAPE\_Sphericity \\ +(-0.02718132)*GLCM\_Homogeneity\_1 \\ +(-0.0000004074912)*GLRLM\_RP\_1 \\ +(-0.0000002804169)*GLRLM\_RP\_2 \\ +(-0.0000002963151)*GLRLM\_RP\_3 \\ +(-0.0000001328383)*GLRLM\_RP\_4 \\ \end{matrix}$$

**Table S1: Used R Packages**

| Package Name | Usage Description |
| --- | --- |
| “glmnet” | Lasso feature selection approach and logistic regression model building  https://cran.r-project.org/web/packages/glmnet/ |
| “Hmisc” | Harrell’s C-Index calculations  https://cran.r-project.org/web/packages/Hmisc/index.html |
| “rms” | Decision curves analysis and calibration curves  https://cran.r-project.org/web/packages/rms/index.html |
| “ggplot2” | General plotting  <https://cran.r-project.org/web/packages/ggplot2/index.html> |
